# 2-Methyl-1*H*-benzimidazol-3-ium hydrogen phthalate

**DOI:** 10.1107/S1600536811036178

**Published:** 2011-09-14

**Authors:** Feng Lin, Shouwen Jin, Kai Tong, Haidong He, YuanQi Yu

**Affiliations:** a Faculty of Science ZheJiang A & F University, Lin’An 311300, People’s Republic of China; b Tianmu college of ZheJiang A & F University, Lin’An 311300, People’s Republic of China

## Abstract

The asymmetric unit of the title compound, C_8_H_9_N_2_^+^·C_8_H_5_O_4_^−^, contains two independent ion pairs. In each 2-methyl-1*H*-benzimidazolium ion, an intra­molecular O—H⋯O bond forms an *S*(7) graph-set motif. In the crystal, the components are linked by N—H⋯O hydrogen bonds, forming chains along [210]. Further stabilization is provided by weak C—H⋯O hydrogen bonds.

## Related literature

For general background to hydrogen-bonding inter­actions, see: Lam & Mak (2000 ▶); Desiraju (2002 ▶); Liu *et al.* (2008 ▶); Biswas *et al.* (2009 ▶); Jin & Wang (2010 ▶). For hydrogen-bond motifs, see: Bernstein *et al.* (1995 ▶).
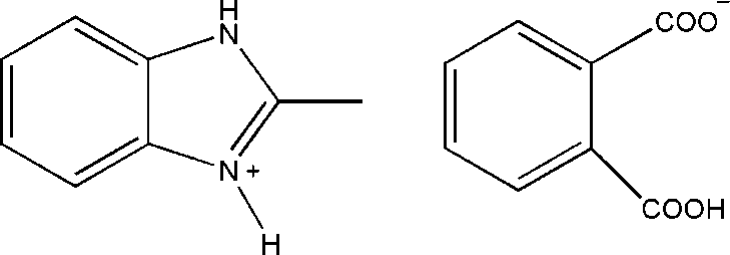


## Experimental

### 

#### Crystal data

C_8_H_9_N_2_^+^·C_8_H_5_O_4_^−^
*M* *_r_* = 298.29
Triclinic, 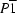

*a* = 3.8545 (16) Å
*b* = 17.689 (7) Å
*c* = 20.752 (9) Å
α = 86.754 (8)°
β = 86.585 (7)°
γ = 84.169 (7)°
*V* = 1403.3 (10) Å^3^
*Z* = 4
Mo *K*α radiation
μ = 0.10 mm^−1^
*T* = 298 K
0.45 × 0.39 × 0.30 mm

#### Data collection

Bruker SMART CCD diffractometer
Absorption correction: multi-scan (*SADABS*; Bruker, 2007 ▶) *T*_min_ = 0.955, *T*_max_ = 0.970
7520 measured reflections
4923 independent reflections
1846 reflections with *I* > 2σ(*I*)
*R*_int_ = 0.058

#### Refinement

*R*[*F* ^2^ > 2σ(*F* ^2^)] = 0.082
*wR*(*F* ^2^) = 0.232
*S* = 0.89
4923 reflections
397 parameters
H-atom parameters constrained
Δρ_max_ = 0.26 e Å^−3^
Δρ_min_ = −0.31 e Å^−3^

### 

Data collection: *SMART* (Bruker, 2007) ▶; cell refinement: *SAINT* (Bruker, 2007) ▶; data reduction: *SAINT* ▶; program(s) used to solve structure: *SHELXS97* (Sheldrick, 2008 ▶); program(s) used to refine structure: *SHELXL97* (Sheldrick, 2008 ▶); molecular graphics: *SHELXTL* (Sheldrick, 2008 ▶) and *Mercury* (Macrae *et al.*, 2006 ▶); software used to prepare material for publication: *SHELXL97*.

## Supplementary Material

Crystal structure: contains datablock(s) global, I. DOI: 10.1107/S1600536811036178/lh5327sup1.cif

Structure factors: contains datablock(s) I. DOI: 10.1107/S1600536811036178/lh5327Isup2.hkl

Supplementary material file. DOI: 10.1107/S1600536811036178/lh5327Isup3.cml

Additional supplementary materials:  crystallographic information; 3D view; checkCIF report

## Figures and Tables

**Table 1:** Hydrogen-bond geometry (Å, °)

*D*—H⋯*A*	*D*—H	H⋯*A*	*D*⋯*A*	*D*—H⋯*A*
N1—H1⋯O4^i^	0.86	1.81	2.649 (6)	166
N2—H2⋯O5^ii^	0.86	1.91	2.750 (6)	165
N2—H2⋯O6^ii^	0.86	2.58	3.226 (6)	133
N3—H3⋯O8^iii^	0.86	1.79	2.631 (6)	166
N4—H4⋯O1^iv^	0.86	1.83	2.680 (6)	168
N4—H4⋯O2^iv^	0.86	2.58	3.232 (6)	133
O3—H3*A*⋯O2	0.82	1.55	2.372 (5)	175
O7—H7⋯O6	0.82	1.56	2.379 (5)	179
C7—H7*A*⋯O6^ii^	0.93	2.53	3.244 (7)	134

Symmetry codes: (i) 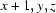; (ii) 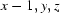; (iii) 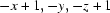; (iv) 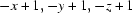.

**Fig. 1.**
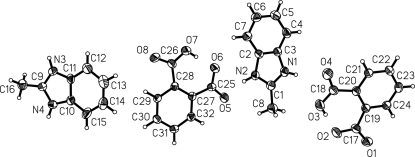
The asymmetric unit of the title compound. Displacement ellipsoids are drawn at the 30% probability level.

**Fig. 2.**
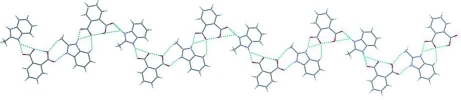
One dimensional chain running along [210] with hydrogen bonds shown as dashed lines.

## supplementary crystallographic information

### Comment

Intermolecular interactions are responsible for crystal packing and gaining an understanding of these interactions allows the comprehension of the collective properties and permits the design of new crystals with specific physical and chemical properties (Lam & Mak, 2000). Hydrogen bonding is one of the most important noncovalent interactions that determines and controls the assembly of molecules and ions (Desiraju, 2002, Liu *et al.*, 2008, Biswas *et al.*, 2009). As an extension of our study concentrating on hydrogen bonded assembly of organic acids and organic bases (Jin *et al.*, 2010), herein we report the crystal structure of the 1:1 salt of 2-methyl-1*H*-benzimidazolium hydrogen phthalate.

The asymmetric unit of the compound consists of two independent 2-methyl-1*H*-benzimidazolium and two independent hydrogen phthalate ions (Fig. 1). Intramolecular hydrogen bonds between the carbonyl groups and the hydroxy groups form S(7) graph motifs (Bernstein *et al.*, 1995). The cations and the anions are connected *via* the N—H···O hydrogen bonds to form one-dimensional chains along [210] (Fig. 2). Further stabilization of the chain is provided by weak intrachain C—H···O hydrogen bonds.

### Experimental

A solution of 2-methyl-1*H*-benzimidazole (13.2 mg, 0.1 mmol) in 3 ml of MeOH was added to a MeOH solution (3 ml) containing phthalic acid acid (16.6 mg, 0.1 mmol) under continuous stirring. The solution was stirred for about 1 h at room temperature, then the solution was filtered into a test tube. The solution was left standing at room temperature for several days, colorless block-shaped crystals were isolated after slow evaporation of the solution in air at ambient temperature.

### Refinement

All H atoms were placed in calculated positions with C—H = 0.93–0.96 Å, N—H = 0.86Å and O—H = 0.82Å and were included in the refinement with *U*_iso_(H) = 1.2*U*_eq_(C,*N*) or *U*_iso_(H) = 1.2*U*_eq_(C_methyl_,*O*).

### Crystal data

**Table:**

C_8_H_9_N_2_^+^·C_8_H_5_O_4_^−^	*Z* = 4
*M**_r_* = 298.29	*F*(000) = 624
Triclinic, *P*1	*D*_x_ = 1.412 Mg m^−^^3^
Hall symbol: -P 1	Mo *K*α radiation, λ = 0.71073 Å
*a* = 3.8545 (16) Å	Cell parameters from 700 reflections
*b* = 17.689 (7) Å	θ = 2.2–19.6°
*c* = 20.752 (9) Å	µ = 0.10 mm^−^^1^
α = 86.754 (8)°	*T* = 298 K
β = 86.585 (7)°	Block, colorless
γ = 84.169 (7)°	0.45 × 0.39 × 0.30 mm
*V* = 1403.3 (10) Å^3^	

### Data collection

**Table:**

Bruker SMART CCD diffractometer	4923 independent reflections
Radiation source: fine-focus sealed tube	1846 reflections with *I* > 2σ(*I*)
graphite	*R*_int_ = 0.058
φ and ω scans	θ_max_ = 25.0°, θ_min_ = 2.2°
Absorption correction: multi-scan (*SADABS*; Bruker, 2007)	*h* = −4→4
*T*_min_ = 0.955, *T*_max_ = 0.970	*k* = −20→19
7520 measured reflections	*l* = −21→24

### Refinement

**Table:**

Refinement on *F*^2^	Primary atom site location: structure-invariant direct methods
Least-squares matrix: full	Secondary atom site location: difference Fourier map
*R*[*F*^2^ > 2σ(*F*^2^)] = 0.082	Hydrogen site location: inferred from neighbouring sites
*wR*(*F*^2^) = 0.232	H-atom parameters constrained
*S* = 0.89	*w* = 1/[σ^2^(*F*_o_^2^) + (0.0958*P*)^2^] where *P* = (*F*_o_^2^ + 2*F*_c_^2^)/3
4923 reflections	(Δ/σ)_max_ < 0.001
397 parameters	Δρ_max_ = 0.26 e Å^−^^3^
0 restraints	Δρ_min_ = −0.31 e Å^−^^3^

### Special details

**Table:**

Geometry. All e.s.d.'s (except the e.s.d. in the dihedral angle between two l.s. planes) are estimated using the full covariance matrix. The cell e.s.d.'s are taken into account individually in the estimation of e.s.d.'s in distances, angles and torsion angles; correlations between e.s.d.'s in cell parameters are only used when they are defined by crystal symmetry. An approximate (isotropic) treatment of cell e.s.d.'s is used for estimating e.s.d.'s involving l.s. planes.
Refinement. Refinement of *F*^2^ against ALL reflections. The weighted *R*-factor *wR* and goodness of fit *S* are based on *F*^2^, conventional *R*-factors *R* are based on *F*, with *F* set to zero for negative *F*^2^. The threshold expression of *F*^2^ > σ(*F*^2^) is used only for calculating *R*-factors(gt) *etc*. and is not relevant to the choice of reflections for refinement. *R*-factors based on *F*^2^ are statistically about twice as large as those based on *F*, and *R*- factors based on ALL data will be even larger.

### Fractional atomic coordinates and isotropic or equivalent isotropic displacement parameters (Å^2^)

**Table:**

	*x*	*y*	*z*	*U*_iso_*/*U*_eq_	
N1	0.6243 (11)	0.5633 (2)	0.1657 (2)	0.0614 (13)	
H1	0.7074	0.6025	0.1465	0.074*	
N2	0.3976 (11)	0.4903 (2)	0.2389 (2)	0.0572 (12)	
H2	0.3100	0.4741	0.2756	0.069*	
N3	0.4721 (12)	−0.0649 (2)	0.6631 (2)	0.0661 (14)	
H3	0.4929	−0.1059	0.6423	0.079*	
N4	0.4893 (12)	0.0117 (3)	0.7390 (2)	0.0615 (13)	
H4	0.5221	0.0285	0.7760	0.074*	
O1	0.3597 (12)	0.9545 (2)	0.14225 (18)	0.0842 (14)	
O2	0.2701 (12)	0.8461 (2)	0.18961 (19)	0.0886 (14)	
O3	0.0573 (12)	0.7292 (2)	0.16867 (19)	0.0829 (13)	
H3A	0.1415	0.7687	0.1753	0.124*	
O4	−0.1224 (12)	0.6721 (2)	0.0893 (2)	0.0863 (14)	
O5	1.0493 (11)	0.4630 (2)	0.35612 (17)	0.0766 (13)	
O6	0.9446 (12)	0.3625 (2)	0.30921 (19)	0.0867 (14)	
O7	0.7319 (12)	0.2419 (2)	0.33335 (19)	0.0838 (13)	
H7	0.8077	0.2833	0.3253	0.126*	
O8	0.5216 (13)	0.1768 (2)	0.4153 (2)	0.0917 (15)	
C1	0.5080 (14)	0.5583 (3)	0.2263 (3)	0.0572 (15)	
C2	0.4456 (15)	0.4496 (3)	0.1839 (3)	0.0561 (14)	
C3	0.5921 (14)	0.4953 (3)	0.1373 (3)	0.0529 (14)	
C4	0.6727 (14)	0.4745 (3)	0.0752 (3)	0.0643 (16)	
H4A	0.7639	0.5075	0.0436	0.077*	
C5	0.6105 (17)	0.4017 (4)	0.0624 (3)	0.0785 (19)	
H5	0.6664	0.3839	0.0214	0.094*	
C6	0.4652 (16)	0.3546 (3)	0.1102 (3)	0.0701 (17)	
H6	0.4202	0.3063	0.0997	0.084*	
C7	0.3858 (14)	0.3762 (3)	0.1717 (3)	0.0623 (16)	
H7A	0.2963	0.3434	0.2036	0.075*	
C8	0.5046 (17)	0.6182 (3)	0.2734 (3)	0.0760 (18)	
H8A	0.2687	0.6396	0.2823	0.114*	
H8B	0.6421	0.6575	0.2556	0.114*	
H8C	0.6008	0.5966	0.3127	0.114*	
C9	0.5569 (14)	−0.0599 (3)	0.7229 (3)	0.0559 (14)	
C10	0.3580 (15)	0.0550 (3)	0.6869 (3)	0.0590 (15)	
C11	0.3466 (14)	0.0050 (3)	0.6392 (3)	0.0575 (15)	
C12	0.2216 (16)	0.0290 (4)	0.5784 (3)	0.0707 (17)	
H12	0.2144	−0.0043	0.5455	0.085*	
C13	0.1106 (17)	0.1053 (4)	0.5709 (3)	0.087 (2)	
H13	0.0230	0.1243	0.5318	0.104*	
C14	0.1258 (16)	0.1536 (4)	0.6194 (4)	0.0769 (19)	
H14	0.0493	0.2048	0.6120	0.092*	
C15	0.2482 (17)	0.1302 (3)	0.6784 (3)	0.0769 (19)	
H15	0.2562	0.1639	0.7110	0.092*	
C16	0.6985 (15)	−0.1238 (3)	0.7661 (3)	0.0714 (17)	
H16A	0.5251	−0.1588	0.7753	0.107*	
H16B	0.7597	−0.1045	0.8058	0.107*	
H16C	0.9025	−0.1496	0.7453	0.107*	
C17	0.2781 (16)	0.8892 (4)	0.1390 (3)	0.0648 (16)	
C18	−0.0014 (16)	0.7282 (3)	0.1088 (3)	0.0624 (16)	
C19	0.1934 (14)	0.8627 (3)	0.0755 (3)	0.0533 (14)	
C20	0.0778 (14)	0.7921 (3)	0.0617 (3)	0.0546 (14)	
C21	0.0164 (15)	0.7799 (3)	−0.0009 (3)	0.0617 (16)	
H21	−0.0674	0.7343	−0.0097	0.074*	
C22	0.0716 (16)	0.8313 (3)	−0.0510 (3)	0.0660 (16)	
H22	0.0319	0.8201	−0.0931	0.079*	
C23	0.1877 (16)	0.9002 (3)	−0.0384 (3)	0.0663 (16)	
H23	0.2251	0.9365	−0.0717	0.080*	
C24	0.2460 (15)	0.9138 (3)	0.0239 (3)	0.0607 (15)	
H24	0.3256	0.9601	0.0321	0.073*	
C25	0.9394 (15)	0.4004 (3)	0.3595 (3)	0.0584 (15)	
C26	0.6405 (16)	0.2354 (3)	0.3930 (3)	0.0621 (16)	
C27	0.7972 (13)	0.3692 (3)	0.4231 (2)	0.0479 (13)	
C28	0.6743 (13)	0.2974 (3)	0.4380 (2)	0.0476 (13)	
C29	0.5680 (13)	0.2802 (3)	0.5007 (3)	0.0493 (13)	
H29	0.4900	0.2327	0.5110	0.059*	
C30	0.5710 (15)	0.3294 (3)	0.5490 (3)	0.0633 (16)	
H30	0.4947	0.3153	0.5909	0.076*	
C31	0.6872 (15)	0.4000 (3)	0.5354 (3)	0.0612 (16)	
H31	0.6900	0.4347	0.5673	0.073*	
C32	0.7995 (14)	0.4170 (3)	0.4723 (3)	0.0562 (15)	
H32	0.8820	0.4642	0.4627	0.067*	

### Atomic displacement parameters (Å^2^)

**Table:**

	*U*^11^	*U*^22^	*U*^33^	*U*^12^	*U*^13^	*U*^23^
N1	0.065 (3)	0.060 (3)	0.059 (3)	−0.019 (2)	0.006 (3)	0.008 (3)
N2	0.070 (3)	0.051 (3)	0.052 (3)	−0.015 (2)	−0.005 (2)	0.005 (2)
N3	0.093 (4)	0.053 (3)	0.058 (3)	−0.028 (3)	−0.011 (3)	−0.007 (3)
N4	0.078 (4)	0.063 (3)	0.048 (3)	−0.020 (3)	−0.010 (3)	−0.008 (3)
O1	0.120 (4)	0.070 (3)	0.070 (3)	−0.035 (3)	−0.013 (3)	−0.017 (2)
O2	0.137 (4)	0.085 (3)	0.049 (3)	−0.033 (3)	−0.016 (3)	0.003 (2)
O3	0.117 (4)	0.077 (3)	0.058 (3)	−0.031 (3)	−0.006 (3)	0.014 (2)
O4	0.121 (4)	0.066 (3)	0.079 (3)	−0.045 (3)	−0.003 (3)	0.005 (2)
O5	0.106 (3)	0.069 (3)	0.060 (3)	−0.043 (3)	0.003 (2)	0.003 (2)
O6	0.130 (4)	0.078 (3)	0.054 (3)	−0.028 (3)	0.015 (3)	−0.004 (2)
O7	0.127 (4)	0.068 (3)	0.062 (3)	−0.034 (3)	0.004 (3)	−0.012 (2)
O8	0.143 (4)	0.061 (3)	0.078 (3)	−0.048 (3)	0.014 (3)	−0.015 (2)
C1	0.054 (4)	0.060 (4)	0.059 (4)	−0.013 (3)	0.001 (3)	−0.002 (3)
C2	0.068 (4)	0.056 (3)	0.045 (3)	−0.013 (3)	−0.007 (3)	−0.002 (3)
C3	0.059 (4)	0.053 (3)	0.047 (4)	−0.010 (3)	−0.002 (3)	−0.001 (3)
C4	0.067 (4)	0.074 (4)	0.049 (4)	−0.002 (3)	0.008 (3)	0.005 (3)
C5	0.098 (5)	0.080 (5)	0.056 (4)	0.001 (4)	−0.004 (4)	−0.011 (4)
C6	0.079 (5)	0.062 (4)	0.072 (5)	−0.007 (3)	−0.017 (4)	−0.011 (4)
C7	0.070 (4)	0.058 (4)	0.061 (4)	−0.017 (3)	−0.003 (3)	−0.005 (3)
C8	0.093 (5)	0.072 (4)	0.065 (4)	−0.019 (4)	−0.005 (4)	−0.007 (3)
C9	0.065 (4)	0.050 (3)	0.054 (4)	−0.012 (3)	−0.004 (3)	−0.008 (3)
C10	0.070 (4)	0.060 (4)	0.053 (4)	−0.034 (3)	−0.009 (3)	0.001 (3)
C11	0.067 (4)	0.062 (4)	0.048 (4)	−0.033 (3)	−0.007 (3)	0.008 (3)
C12	0.070 (4)	0.082 (5)	0.063 (4)	−0.026 (4)	−0.008 (3)	0.001 (4)
C13	0.081 (5)	0.106 (6)	0.077 (5)	−0.030 (5)	−0.016 (4)	0.020 (5)
C14	0.065 (4)	0.067 (4)	0.093 (5)	0.001 (3)	0.000 (4)	0.020 (4)
C15	0.088 (5)	0.052 (4)	0.093 (5)	−0.026 (3)	−0.004 (4)	0.002 (4)
C16	0.069 (4)	0.074 (4)	0.071 (4)	−0.009 (3)	−0.008 (3)	0.003 (3)
C17	0.079 (5)	0.069 (4)	0.050 (4)	−0.023 (3)	−0.003 (3)	−0.006 (3)
C18	0.075 (4)	0.063 (4)	0.051 (4)	−0.016 (3)	−0.006 (3)	−0.002 (3)
C19	0.063 (4)	0.049 (3)	0.050 (4)	−0.016 (3)	−0.005 (3)	−0.003 (3)
C20	0.067 (4)	0.045 (3)	0.054 (4)	−0.017 (3)	−0.003 (3)	0.004 (3)
C21	0.090 (5)	0.048 (3)	0.052 (4)	−0.022 (3)	−0.015 (3)	−0.002 (3)
C22	0.090 (5)	0.064 (4)	0.045 (4)	−0.011 (3)	−0.008 (3)	0.000 (3)
C23	0.088 (5)	0.053 (4)	0.058 (4)	−0.018 (3)	0.003 (3)	0.012 (3)
C24	0.085 (4)	0.044 (3)	0.056 (4)	−0.022 (3)	−0.007 (3)	−0.002 (3)
C25	0.071 (4)	0.053 (4)	0.054 (4)	−0.013 (3)	−0.006 (3)	−0.010 (3)
C26	0.077 (4)	0.068 (4)	0.043 (4)	−0.017 (3)	−0.006 (3)	−0.001 (3)
C27	0.046 (3)	0.049 (3)	0.049 (3)	−0.007 (3)	−0.007 (3)	0.001 (3)
C28	0.047 (3)	0.046 (3)	0.051 (3)	−0.010 (2)	−0.001 (3)	0.001 (3)
C29	0.052 (3)	0.041 (3)	0.055 (4)	−0.005 (2)	−0.001 (3)	−0.001 (3)
C30	0.089 (5)	0.062 (4)	0.042 (3)	−0.023 (3)	−0.001 (3)	−0.001 (3)
C31	0.082 (4)	0.051 (3)	0.054 (4)	−0.016 (3)	−0.002 (3)	−0.012 (3)
C32	0.066 (4)	0.049 (3)	0.057 (4)	−0.017 (3)	−0.009 (3)	0.001 (3)

### Geometric parameters (Å, °)

**Table:**

N1—C1	1.312 (6)	C10—C15	1.360 (7)
N1—C3	1.389 (6)	C10—C11	1.370 (7)
N1—H1	0.8600	C11—C12	1.404 (7)
N2—C1	1.323 (6)	C12—C13	1.376 (8)
N2—C2	1.376 (6)	C12—H12	0.9300
N2—H2	0.8600	C13—C14	1.365 (8)
N3—C9	1.312 (6)	C13—H13	0.9300
N3—C11	1.358 (6)	C14—C15	1.365 (8)
N3—H3	0.8600	C14—H14	0.9300
N4—C9	1.323 (6)	C15—H15	0.9300
N4—C10	1.380 (6)	C16—H16A	0.9600
N4—H4	0.8600	C16—H16B	0.9600
O1—C17	1.234 (6)	C16—H16C	0.9600
O2—C17	1.263 (6)	C17—C19	1.487 (7)
O3—C18	1.278 (6)	C18—C20	1.494 (7)
O3—H3A	0.8200	C19—C24	1.380 (7)
O4—C18	1.237 (6)	C19—C20	1.418 (6)
O5—C25	1.222 (6)	C20—C21	1.366 (7)
O6—C25	1.271 (6)	C21—C22	1.364 (7)
O7—C26	1.269 (6)	C21—H21	0.9300
O7—H7	0.8200	C22—C23	1.385 (7)
O8—C26	1.230 (6)	C22—H22	0.9300
C1—C8	1.480 (7)	C23—C24	1.365 (7)
C2—C3	1.359 (7)	C23—H23	0.9300
C2—C7	1.382 (7)	C24—H24	0.9300
C3—C4	1.369 (7)	C25—C27	1.496 (7)
C4—C5	1.377 (7)	C26—C28	1.500 (7)
C4—H4A	0.9300	C27—C32	1.362 (6)
C5—C6	1.386 (8)	C27—C28	1.411 (6)
C5—H5	0.9300	C28—C29	1.369 (7)
C6—C7	1.361 (7)	C29—C30	1.364 (6)
C6—H6	0.9300	C29—H29	0.9300
C7—H7A	0.9300	C30—C31	1.377 (6)
C8—H8A	0.9600	C30—H30	0.9300
C8—H8B	0.9600	C31—C32	1.381 (7)
C8—H8C	0.9600	C31—H31	0.9300
C9—C16	1.483 (7)	C32—H32	0.9300
			
C1—N1—C3	109.2 (5)	C15—C14—H14	118.5
C1—N1—H1	125.4	C10—C15—C14	116.4 (6)
C3—N1—H1	125.4	C10—C15—H15	121.8
C1—N2—C2	109.1 (4)	C14—C15—H15	121.8
C1—N2—H2	125.4	C9—C16—H16A	109.5
C2—N2—H2	125.4	C9—C16—H16B	109.5
C9—N3—C11	109.3 (4)	H16A—C16—H16B	109.5
C9—N3—H3	125.3	C9—C16—H16C	109.5
C11—N3—H3	125.3	H16A—C16—H16C	109.5
C9—N4—C10	109.2 (4)	H16B—C16—H16C	109.5
C9—N4—H4	125.4	O1—C17—O2	119.2 (5)
C10—N4—H4	125.4	O1—C17—C19	119.4 (5)
C18—O3—H3A	109.5	O2—C17—C19	121.5 (5)
C26—O7—H7	109.5	O4—C18—O3	118.7 (5)
N1—C1—N2	108.8 (5)	O4—C18—C20	119.3 (5)
N1—C1—C8	125.8 (5)	O3—C18—C20	122.0 (5)
N2—C1—C8	125.4 (5)	C24—C19—C20	117.2 (5)
C3—C2—N2	106.8 (5)	C24—C19—C17	114.3 (5)
C3—C2—C7	120.8 (5)	C20—C19—C17	128.4 (5)
N2—C2—C7	132.4 (5)	C21—C20—C19	118.3 (5)
C2—C3—C4	123.6 (5)	C21—C20—C18	114.3 (5)
C2—C3—N1	106.2 (5)	C19—C20—C18	127.4 (5)
C4—C3—N1	130.2 (5)	C22—C21—C20	123.4 (5)
C3—C4—C5	115.8 (5)	C22—C21—H21	118.3
C3—C4—H4A	122.1	C20—C21—H21	118.3
C5—C4—H4A	122.1	C21—C22—C23	119.0 (5)
C4—C5—C6	120.6 (6)	C21—C22—H22	120.5
C4—C5—H5	119.7	C23—C22—H22	120.5
C6—C5—H5	119.7	C24—C23—C22	118.5 (5)
C7—C6—C5	122.8 (6)	C24—C23—H23	120.7
C7—C6—H6	118.6	C22—C23—H23	120.7
C5—C6—H6	118.6	C23—C24—C19	123.6 (5)
C6—C7—C2	116.2 (5)	C23—C24—H24	118.2
C6—C7—H7A	121.9	C19—C24—H24	118.2
C2—C7—H7A	121.9	O5—C25—O6	119.9 (6)
C1—C8—H8A	109.5	O5—C25—C27	119.6 (5)
C1—C8—H8B	109.5	O6—C25—C27	120.5 (5)
H8A—C8—H8B	109.5	O8—C26—O7	119.9 (5)
C1—C8—H8C	109.5	O8—C26—C28	118.4 (5)
H8A—C8—H8C	109.5	O7—C26—C28	121.8 (5)
H8B—C8—H8C	109.5	C32—C27—C28	117.7 (5)
N3—C9—N4	108.7 (5)	C32—C27—C25	113.9 (5)
N3—C9—C16	125.9 (5)	C28—C27—C25	128.4 (5)
N4—C9—C16	125.4 (5)	C29—C28—C27	118.0 (5)
C15—C10—C11	122.2 (6)	C29—C28—C26	113.9 (5)
C15—C10—N4	132.4 (5)	C27—C28—C26	128.2 (5)
C11—C10—N4	105.4 (5)	C30—C29—C28	123.2 (5)
N3—C11—C10	107.4 (5)	C30—C29—H29	118.4
N3—C11—C12	131.1 (5)	C28—C29—H29	118.4
C10—C11—C12	121.5 (6)	C29—C30—C31	119.7 (5)
C13—C12—C11	115.4 (6)	C29—C30—H30	120.1
C13—C12—H12	122.3	C31—C30—H30	120.1
C11—C12—H12	122.3	C30—C31—C32	117.2 (5)
C14—C13—C12	121.6 (6)	C30—C31—H31	121.4
C14—C13—H13	119.2	C32—C31—H31	121.4
C12—C13—H13	119.2	C27—C32—C31	124.2 (5)
C13—C14—C15	122.9 (6)	C27—C32—H32	117.9
C13—C14—H14	118.5	C31—C32—H32	117.9
			
C3—N1—C1—N2	−0.6 (6)	O1—C17—C19—C24	5.5 (8)
C3—N1—C1—C8	178.7 (5)	O2—C17—C19—C24	−174.2 (6)
C2—N2—C1—N1	0.1 (6)	O1—C17—C19—C20	−176.5 (6)
C2—N2—C1—C8	−179.1 (5)	O2—C17—C19—C20	3.8 (10)
C1—N2—C2—C3	0.4 (6)	C24—C19—C20—C21	−1.9 (8)
C1—N2—C2—C7	177.6 (6)	C17—C19—C20—C21	−179.8 (6)
N2—C2—C3—C4	−178.8 (5)	C24—C19—C20—C18	−179.2 (5)
C7—C2—C3—C4	3.6 (8)	C17—C19—C20—C18	2.9 (9)
N2—C2—C3—N1	−0.7 (6)	O4—C18—C20—C21	−2.2 (8)
C7—C2—C3—N1	−178.3 (5)	O3—C18—C20—C21	176.7 (6)
C1—N1—C3—C2	0.8 (6)	O4—C18—C20—C19	175.3 (6)
C1—N1—C3—C4	178.8 (6)	O3—C18—C20—C19	−5.9 (9)
C2—C3—C4—C5	−2.7 (8)	C19—C20—C21—C22	2.3 (9)
N1—C3—C4—C5	179.6 (5)	C18—C20—C21—C22	179.9 (6)
C3—C4—C5—C6	1.8 (8)	C20—C21—C22—C23	−1.6 (9)
C4—C5—C6—C7	−1.8 (9)	C21—C22—C23—C24	0.7 (9)
C5—C6—C7—C2	2.4 (8)	C22—C23—C24—C19	−0.5 (9)
C3—C2—C7—C6	−3.2 (8)	C20—C19—C24—C23	1.1 (9)
N2—C2—C7—C6	179.9 (6)	C17—C19—C24—C23	179.3 (5)
C11—N3—C9—N4	0.1 (6)	O5—C25—C27—C32	1.3 (7)
C11—N3—C9—C16	179.0 (5)	O6—C25—C27—C32	−178.2 (5)
C10—N4—C9—N3	−0.6 (6)	O5—C25—C27—C28	−176.7 (5)
C10—N4—C9—C16	−179.5 (5)	O6—C25—C27—C28	3.7 (8)
C9—N4—C10—C15	179.7 (6)	C32—C27—C28—C29	−0.5 (7)
C9—N4—C10—C11	0.8 (6)	C25—C27—C28—C29	177.5 (5)
C9—N3—C11—C10	0.4 (6)	C32—C27—C28—C26	179.1 (5)
C9—N3—C11—C12	−179.6 (6)	C25—C27—C28—C26	−2.9 (9)
C15—C10—C11—N3	−179.7 (5)	O8—C26—C28—C29	0.7 (7)
N4—C10—C11—N3	−0.7 (6)	O7—C26—C28—C29	−179.1 (5)
C15—C10—C11—C12	0.3 (9)	O8—C26—C28—C27	−178.9 (5)
N4—C10—C11—C12	179.3 (5)	O7—C26—C28—C27	1.3 (9)
N3—C11—C12—C13	179.5 (6)	C27—C28—C29—C30	1.0 (8)
C10—C11—C12—C13	−0.5 (9)	C26—C28—C29—C30	−178.7 (5)
C11—C12—C13—C14	0.6 (9)	C28—C29—C30—C31	−0.4 (8)
C12—C13—C14—C15	−0.5 (10)	C29—C30—C31—C32	−0.5 (8)
C11—C10—C15—C14	−0.1 (9)	C28—C27—C32—C31	−0.5 (8)
N4—C10—C15—C14	−178.8 (6)	C25—C27—C32—C31	−178.8 (5)
C13—C14—C15—C10	0.2 (9)	C30—C31—C32—C27	1.0 (8)

### Hydrogen-bond geometry (Å, °)

**Table:**

*D*—H···*A*	*D*—H	H···*A*	*D*···*A*	*D*—H···*A*
N1—H1···O4^i^	0.86	1.81	2.649 (6)	166.
N2—H2···O5^ii^	0.86	1.91	2.750 (6)	165.
N2—H2···O6^ii^	0.86	2.58	3.226 (6)	133.
N3—H3···O8^iii^	0.86	1.79	2.631 (6)	166.
N4—H4···O1^iv^	0.86	1.83	2.680 (6)	168.
N4—H4···O2^iv^	0.86	2.58	3.232 (6)	133.
O3—H3A···O2	0.82	1.55	2.372 (5)	175.
O7—H7···O6	0.82	1.56	2.379 (5)	179.
C7—H7A···O6^ii^	0.93	2.53	3.244 (7)	134

Symmetry codes: (i) *x*+1, *y*, *z*; (ii) *x*−1, *y*, *z*; (iii) −*x*+1, −*y*, −*z*+1; (iv) −*x*+1, −*y*+1, −*z*+1.
